# Supplementary material for: A Critical View of the Application of the APEX Software (Aqueous Photochemistry of Environmentally-Occurring Xenobiotics) to Predict Photoreaction Kinetics in Surface Freshwaters
Source: Molecules. 2019 Dec 18;25(1):9. doi: 10.3390/molecules25010009 (PMC7017383; doi:10.3390/molecules25010009)
Supplement: Supplementary file 1 [file molecules-25-00009-s001.zip › 3.2.4_gcc-4.4.0.zip!/3.2.4_gcc-4.4.0/3.2.4_gcc-4.4.0/doc/octave/html/interpreter/Password-Database-Functions.html]

Password Database Functions - Untitled


Next: Group Database Functions,
Previous: Current Working Directory,
Up: System Utilities


---

### 34.9 Password Database Functions

Octave's password database functions return information in a structure
with the following fields.

`name`: The user name. `passwd`: The encrypted password, if available. `uid`: The numeric user id. `gid`: The numeric group id. `gecos`: The GECOS field. `dir`: The home directory. `shell`: The initial shell.

In the descriptions of the following functions, this data structure is
referred to as a pw\_struct.

— Loadable Function: pw\_struct = **getpwent** ()  
> Return a structure containing an entry from the password database,
> opening it if necessary. Once the end of the data has been reached,
> `getpwent` returns 0.

— Loadable Function: pw\_struct = **getpwuid** (uid).  
> Return a structure containing the first entry from the password database
> with the user ID uid. If the user ID does not exist in the
> database, `getpwuid` returns 0.

— Loadable Function: pw\_struct = **getpwnam** (name)  
> Return a structure containing the first entry from the password database
> with the user name name. If the user name does not exist in the
> database, `getpwname` returns 0.

— Loadable Function: **setpwent** ()  
> Return the internal pointer to the beginning of the password database.

— Loadable Function: **endpwent** ()  
> Close the password database.
